# *Lecanicillium aphanocladii* : a biocontrol agent against insect pests and phytopathogens

**DOI:** 10.1002/ps.70621

**Published:** 2026-02-09

**Authors:** Qianhe Liu, Richard Johnson, Kwasi Adusei‐Fosu

**Affiliations:** ^1^ AgResearch Group, Bioeconomy Science Institute Grasslands Research Centre Palmerston North New Zealand

**Keywords:** *Lecanicillium* spp., entomopathogenic fungi, plant protection, biological control agent

## Abstract

Fungi in the genus *Lecanicillium* include a group of important entomopathogenic species affecting insect pests and phytopathogens. *Lecanicillium aphanocladii*, one of 21 recognised *Lecanicillium* species, has been recently reported as a potential biocontrol agent in agricultural and forestry ecosystems with more than 95 *L. aphanocladii* strains having been recorded globally. *Lecanicillium aphanocladii* is a cosmopolitan fungus capable of infecting a broad range of insects, plants and other fungal species. As interest grows in alternative environmentally sustainable insect and plant disease control tools, *L. aphanocladii* appears to be a promising biocontrol option for the agricultural industry. In this review, we highlight recent research findings on the potential of *L. aphanocladii* against insect pests and we provide insights into possible modes of action. Additionally, we review the parasitic nature of *L. aphanocladii* against plant diseases. © 2026 The Author(s). *Pest Management Science* published by John Wiley & Sons Ltd on behalf of Society of Chemical Industry.

## Graphical abstract

Recent research findings on the biocontrol potential of *Lecanicillium aphanocladii* fungus against insect‐pests and plant diseases were highlighted. This review indicates that several *L. aphanocladii* strains show great potential to be developed as multipurpose biocontrol agents active against several insect‐pests, plant diseases and plant parasitic nematodes.

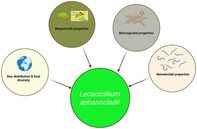

## 1 INTRODUCTION

Globally, crop losses caused by insect pests range from 18% to 26% of annual production, totalling US$470 billion per year.^1^, ^2^ The use of entomopathogens as biological control agents is becoming important, due to negative effects on the environment and non‐target organisms, including mammals, from synthetic chemicals currently used for insect pest control.^1^ Additionally, there is an increasing prevalence of resistance of some pests to these synthetic chemicals and evidence of secondary pest resurgence.

Entomopathogenic fungi are widely used as biopesticides and are expected to have an increasing role in the future. Worldwide, it has been reported that over 700 fungal species belonging to approximately 90 genera are pathogenic to insects.^1^, ^3^, ^4^ Mitosporic fungi such as *Beauveria* spp., *Metarhizium* spp., *Lecanicillium* spp., and *Isaria* spp. (previously *Paecilomyces* spp.) are well known entomopathogens that have been developed commercially as biopesticides.^5^, ^6^, ^7^, ^8^, ^9^ The use and management of these pathogens as biological control agents has been the subject of several major reviews.^1^, ^3^, ^7^, ^10^, ^11^, ^12^, ^13^, ^14^, ^15^, ^16^, ^17^, ^18^, ^19^, ^20^

Fungi in the genus *Lecanicillium* are a group of important insect pathogens.^6^ *Lecanicillium* is in the order Hypocreales and is described as anamorphic Cordycipitaceae. Some *Lecanicillium* species are widely known entomopathogens, such as *Lecanicillium lecanii* (previously named *Verticillium lecanii*). There are up to 21 species currently described in the *Lecanicillium* genus,^21^ including *L. lecanii, Lecanicillium aphanocladii*, *Lecanicillium attenuatum*, *Lecanicillium longisporum, Lecanicillium muscarium*, *Lecanicillium nodulosum* and *Lecanicillium psalliotae*.^22^ These species have been isolated from various hosts exhibiting a wide host range^22^ and have been comprehensively reviewed.^6^, ^7^, ^13^, ^23^ There are at least 26 products based upon *Lecanicillium* species or strains that have been, or are in the process of being developed commercially as biopesticides targeting a variety of pests across several countries.^6^, ^7^, ^24^, ^25^

Recently, the entomopathogenic fungus *L. aphanocladii* has been considered as a potential biocontrol agent for both agricultural and forestry ecosystems. To date, more than 95 *L. aphanocladii* strains are reported in either peer reviewed publications or in the National Centre for Biotechnology Information (NCBI) database (https://www.ncbi.nlm.nih.gov/), based upon GenBank sequencing data. Some strains are reported as control agents of plant pests and have activity against the horse‐chestnut leaf miner moth *Cameraria ohridella* and the lime leaf miner moth *Phyllonorycter* (*Lithocolletis*) *issikii*,^26^, ^27^ aphids *Rhopalosiphum* spp.,^28^ the flour moth *Ephestia kuehniella*,^29^ and the meadow spittlebug *Philaenus spumarius*.^30^ *Lecanicillium aphanocladii* is also a fungicolous species that feeds on *Agaricus bisporus*, *Agaricus bitorquis*, the powdery mildew *Podosphaera* (*Erysiphe*) *fuliginea*, *Podosphaera pannosa*, and the rusts *Puccinia* spp. and *Uromyces savulescui*.^31^

**Figure 1:**
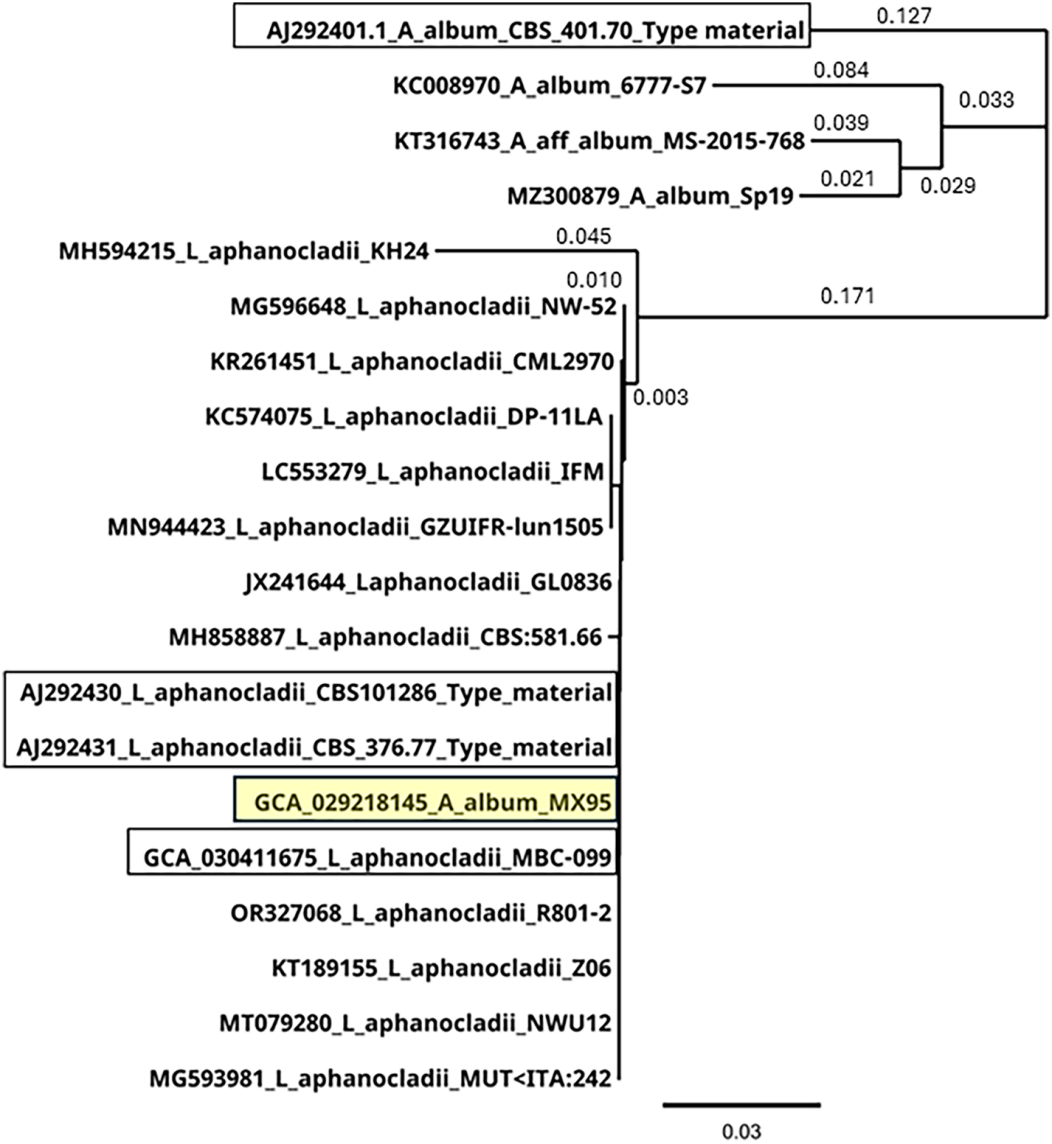
A phylogenetic analysis of internal transcribed spacer (ITS) sequences of selected strains of *Lecanicillium aphanocladii* and *Aphanocladium album*. Sequences were trimmed to match ITS length of the Type strain of *L. aphanocladii* AJ292430 prior to producing the phylogenetic tree. The phylogenetic tree was created by Geneious Tree Builder using the Neighbour‐Joining tree building method and the Tamura‐Nei model.

**Figure 2:**
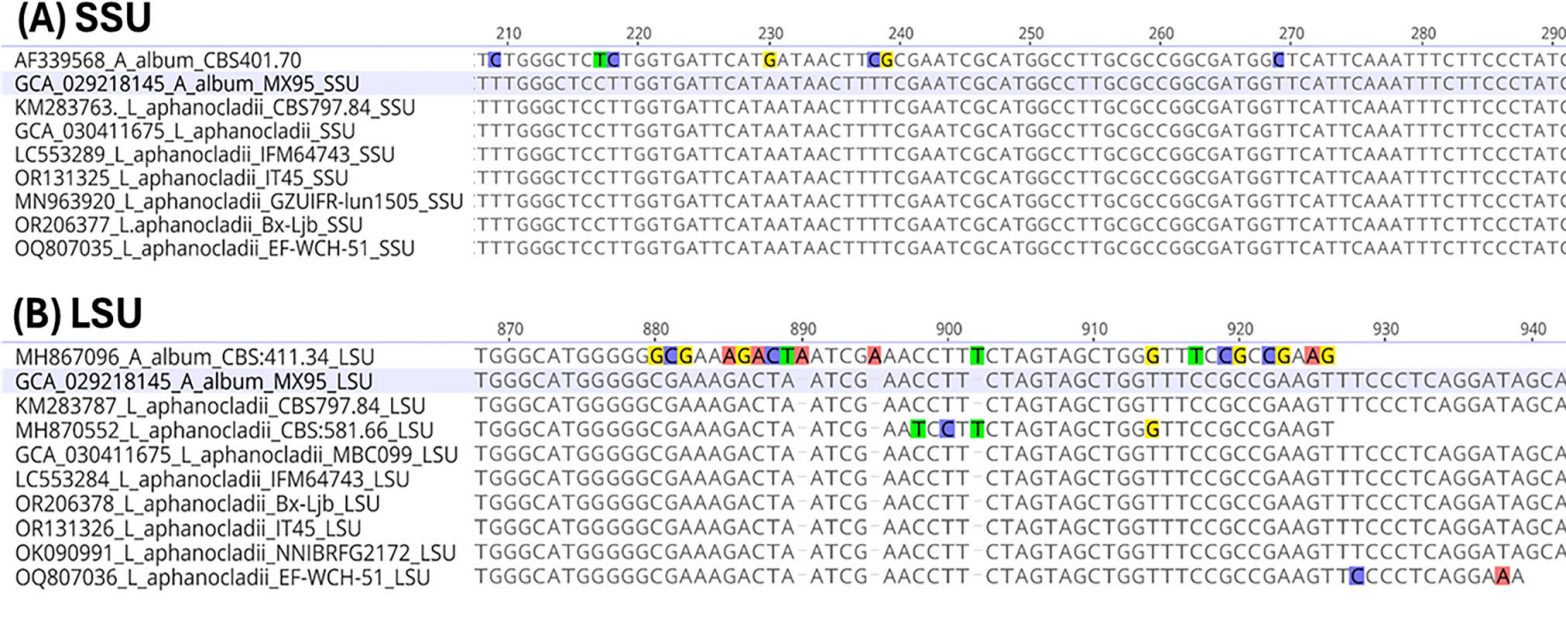
Comparison of partial sequences of small‐subunit rRNA (SSU) (A) and large‐subunit rRNA (LSU) (B) in *Aphanocladium album* strain MX95 with those in known *Lecanicillium aphanocladii* strains. SSU and LSU sequences of *Aphanocladium album* MX95 and *L. aphanocladii* MBC 099 were extracted from genome assemblies of GCA_0.29218145 and GCA_030411675, respectively. The remaining sequences were selected from GenBank with the Accession numbers listed.

Published evidence would suggest there is one registered product named *L. aphanocladii‐*Hu 1 that has been patented in China as a biocontrol agent against green peach aphid *Myzus persicae* (Sulzer) (CN119320701A^32^). A product based on a fungus described as *Aphanocladium album* (Preuss) W. Gams MX95 was also claimed to be a biocontrol agent (patent MI2006A000503; also see D'Ambrosio *et al*.^33^). However, molecular studies have shown that the *Aphanocladium album* MX95 is likely a strain of *L. aphanocladii* (see Section 3). Therefore, in this present review the former name, *Aphanocladium album* MX95, is referred to as *L. aphanocladii* MX95 to avoid confusion unless the name of *Aphanocladium album* is specified (e.g., in Figs 1 and 2).

## 2 LITERATURE SEARCH APPROACH AND SOURCE OF INFORMATION

An analysis of publications and patents available from online platforms was performed using Google Scholar, Scopus, Web of Science, CAB abstracts, CABI Digital Library, PubMed, Food Science and Technology Abstracts, BIOSIS Previews, NCBI, and five patent search engines and databases including Google Patents, Lens, Espacenet, Patentscope, and the United States Patent and Trademark Office (USPTO). There were 11 keywords used for retrieving information ‘*Lecanicillium spp*.’, ‘*Lecanicillium aphanocladii*’, ‘*L. aphanocladii*’, ‘*Aphanocladium album*’, ‘*A. album*’, ‘MX95’, ‘MX‐95’, ‘*Aphanocladium aranearum’, ‘Acremonium aranearum*’, *‘A. aranearum*’, and ‘entomopathogenic fungi’.

## 3 RENAMING OF *APHANOCLADIUM ALBUM* MX95

*Lecanicillium aphanocladii* was previously called *Aphanocladium aranearum* and *Acremonium aranearum* and was first described by Zare and Gams^22^ as a fungicolous fungus. Historically, many *L. aphanocladii* strains had been misidentified as *Aphanocladium album*,^34^ for example, strain ARSEF 6433^35^, ^36^, ^37^ (see more misidentified strains at Table 1). The generic name *Aphanocladium album* is linked to *Aphanocladium album sensu stricto*, whose phylogenetic position is not yet resolved.^22^ Generally, *Aphanocladium album sensu stricto* grows more slowly and is known only as a parasite of slime moulds, while *L. aphanocladii* grows rapidly^22^ and most of its strains produce red pigments in agar.^22^, ^68^ The morphological and molecular characteristics of *L. aphanocladii* and *Aphanocladium album* have been documented.^22^, ^30^, ^31^, ^76^

**Table 1:** Strains of *Lecanicillium aphanocladii* collected from NCBI GenBank and published articles. Same strains may vary in strain ID depending on the collection. ITS, SSU, LSU and Tef1α in National Centre for Biotechnology Information (NCBI) accession represent the gene sequences of internal transcribed spacer, small‐subunit rRNA, large‐subunit rRNA and translation elongation factor 1‐alpha, respectively.

Strain	Name change	NCBI accession	Host/source	Country	Reference
*Lecanicillium aphanocladii CEP556*			Insect (*Araneae* spp.)	Argentina	^38^
*Lecanicillium aphanocladii*			Insect (*Aphididae* sp.)	Iran	^28^
*Lecanicillium aphanocladii* MBC 100		GCA_030411675	Insect (*Aphididae* sp.)	USA	GenBank
*Lecanicillium aphanocladii clone* DP‐11LA		KC574075_ITS	Insect (*Cameraria ohridella; Phyllonorycter issikii*)	Lithuania	^26^, ^27^
*Lecanicillium aphanocladii* GMSL58A		LT220793_Tef1α	Insect (*Galleria mellonella*)	Portugal	GenBank
*Lecanicillium aphanocladii* DSF			Insect (*Philaenus spumarius*)	Italy	^30^
*Lecanicillium aphanocladii*		OP321409_ITS	Insect (*Monochamus alternatus*)	China	^39^
*Lecanicillium aphanocladii* LPV‐3		KY433313_ITS	Insect (*Pseudips mexicanus*)	Mexico	^40^
*Lecanicillium aphanocladii* F28’			Insect (*Ephestia kuehniella*)	Tunisian	^29^
*Lecanicillium aphanocladii* R801‐2 (IRAN‐3692 C)		OR327068_ITS	Insect (*Tetranychus urtica*)	Iran	^41^
*Lecanicillium aphanocladii*			Insect (*Melolontha melolontha*)	Switzerland	^42^
*Lecanicillium aphanocladii*			Cyst nematode (*Globodera* sp.)	Iran	^43^
*Lecanicillium aphanocladii* AF15		PP380387_ITS	Plant (*Astragalus* sp.)	China	^44^
*Lecanicillium aphanocladii* CBS 274.76	*Aphanocladium aranearum Aphanocladium album*		Plant (*Abelmoschus esculentus*)	Bulgaria	^22^
*Lecanicillium aphanocladii* CBS 581.66	*Aphanocladium album*	MH858887_ITS MH870552_LSU	Plant (*Acacia karroo*)	South Africa	^22^
*Lecanicillium aphanocladii* NW‐52		MG596648_ITS	Plant (*Anacardium occidentale* nut)	South Africa	GenBank
*Lecanicillium aphanocladii*	*Aphanocladium aranearum*		Plant (*Eusideroxylon zwageri*)	Malaysia	^45^
*Lecanicillium aphanocladii*			Plant (*Pinus greggii*)	Mexico	^46^
*Lecanicillium aphanocladii*			Plant (*Hordeum vulgare*)	Kazakhstan	^47^
*Lecanicillium aphanocladii* NWU12		MT079280_ITS	Plant (*Arachis hypogaea*)	South Africa	^48^
*Lecanicillium aphanocladii* NWUSeq12		MK841424_ITS	Plant (*Arachis hypogaea*)	South Africa	GenBank
*Lecanicillium aphanocladii* BC stem cutting 2		MW130744_ITS	Plant (*Cannabis sativa* stem)	Canada	GenBank
*Lecanicillium aphanocladii* stems		MT311365_ITS	Plant (*Cannabis sativa* stem)	Canada	GenBank
*Lecanicillium aphanocladii* PDD42022			Plant (*Cyphomandra betacea*)	New Zealand	Landcare Research
*Lecanicillium aphanocladii* IHEM:03304		OW983117_ITS	Plant (*Fagus sylvatica*)	Belgium	GenBank
*Lecanicillium aphanocladii* IHEM:3234		OW983089_ITS	Plant (*Fagus sylvatica*)	Belgium	GenBank
*Lecanicillium aphanocladii* FL17		KP689173‐ITS	Plant (*Huperzia serrata*)	China	^49^
*Lecanicillium aphanocladii* GL0836		JX241644_ITS	Plant (*Malus pumila*)	China	^50^
*Lecanicillium aphanocladii* FJTA		KJ495738‐ITS	Plant (*Nicotiana tabacum*)	China	GenBank
*Lecanicillium aphanocladii* FJTL		KJ162356_ITS	Plant (*Nicotiana tabacum*)	China	GenBank
*Lecanicillium aphanocladii* FF31		KR912316_ITS	Plant (*Pyrus communis* fruit)	South Africa	GenBank
*Lecanicillium aphanocladii* MX95	*Aphanocladium album* MX95	GCA_029218145	Plant (*Solanum melongena*)	Italia	GenBank
*Lecanicillium aphanocladii* IT45		OR131317_ITS OR131325_SSU OR131326_LSU	Plant (*Solanum torvum* seed)	Italy	GenBank
*Lecanicillium aphanocladii* SGP‐4			Plant (*Morus alba*)	China	^51^
*Lecanicillium aphanocladii* N11		MK304173_ITS	Plant endophyte (*Ageratina adenophora*)	China	GenBank
*Lecanicillium aphanocladii* KH24		MH594215_ITS	Plant endophyte (*Hemidesmus indicus*)	India	^52^
*Lecanicillium aphanocladii* N15		MK304090_ITS	Plant endophyte (*Ageratina adenophora*)	China	GenBank
*Lecanicillium aphanocladii* N63		MK304418_ITS	Plant endophyte (*Ageratina adenophora*)	China	GenBank
*Lecanicillium aphanocladii* FS13		KP689216_ITS	Plant endophyte (*Huperzia serrata*)	China	^49^
*Lecanicillium aphanocladii* BLH_238		OM436882_ITS	Plant endophyte (*Macleaya cordata*)	China	GenBank
*Lecanicillium aphanocladii* EF‐WCH‐51		OQ806916_ITS OQ807035_SSU OQ807036_LSU OQ857508_Tef1α	Plant endophyte (*Magnolia denudata* cv. *Hailuo*)	China	^53^
*Lecanicillium aphanocladii* AAH4‐1		OQ162343_ITS	Plant endophyte (*Physalis peruviana* seed)	Iran	GenBank
*Lecanicillium aphanocladii*			Plant endophyte (*Picea mariana*)	Canada	^54^
*Lecanicillium aphanocladii* BCR‐427	*Aphanocladium aranearum*	ON740940_ITS	Plant endophyte (*Bupleurum chinense*)	China	^55^
*Lecanicillium aphanocladii* voucher EXF‐2447			Plant endophyte (*Zea mays* W22 seed)	Slovenia	^56^
*Lecanicillium aphanocladii* PDD42012			Fungi (*Altenaria tenius*)	New Zealand	Landcare Research
*Lecanicillium aphanocladii* CBS 376.77	*Aphanocladium aranearum*	AJ292431 _ITS	Mushroom fungi (*Agaricus bitorquis*)	The Netherlands	^30^
*Lecanicillium aphanocladii* CBS 774.83	*Aphanocladium album*		Mushroom fungi (*Agaricus bitorquis*)	Unknown	^22^
*Lecanicillium aphanocladii* CBS 797.84	*Aphanocladium aranearum Aphanocladium album*	KM283763_SSU KM283787_LSU KM283811_Tef1α	Mushroom fungi (*Agaricus bitorquis*)	China	^22^
*Lecanicillium aphanocladii* CBS 798.84	*Aphanocladium aranearum Aphanocladium album*		Mushroom fungi (*Agaricus bitorquis*)	China	^22^
*Lecanicillium aphanocladii* CBS 101286	*Aphanocladium aranearum* IMI 96000b *Verticillium psalliotae*	AJ292430_ITS	Mushroom fungi (*Agaricus bitorquis*)	UK	GenBank
*Lecanicillium aphanocladii* G1		OL629617‐ITS	Mushroom fungi (*Morchella* spp.)	China	^57^
*Lecanicillium aphanocladii* JAUCC5994		OR225843_ITS OR333513_LSU	Mushroom fungi (*Schizophyllum commune*)	China	^58^
*Lecanicillium aphanocladii*		KX530079_ITS	Mushrrom fungi (*Tremella fuciformis*)	China	^59^
*Lecanicillium aphanocladii* Bx‐Ljb		OR077442_ITS OR206377_SSU OR206378_LSU OR204702_Tef1α	Mushroom fungi (*Sparassis latifolia*)	China	^60^
*Lecanicillium aphanocladii* GMSL01		LT220701_ITS LT220793_Tef1α	Vineyard soil	Portugal	^61^
*Lecanicillium aphanocladii* GMSL02		LT220702_ITS	Vineyard soil	Portugal	^61^
*Lecanicillium aphanocladii* GZUIFR‐lun1505		MN944423_ITS MN963920_SSU	Forest soil	China	^62^
*Lecanicillium aphanocladii* ZNF15		OP379501_ITS	Rhizosphere soil of *Astragalus forrestii*	China	^63^
*Lecanicillium aphanocladii* GZUIFR.SP477		KX021371_ITS	Wheat soil	China	GenBank
Lecanicillium aphanocladii CML2970			Cave soil	Brazil	^64^
Lecanicillium aphanocladii CML2971			Soil	Iraq	^65^
*Lecanicillium aphanocladii* MUT<ITA>:242		MG593981_ITS	Oil contaminated soil	Russia	^66^, ^67^
*Lecanicillium aphanocladii* YARK7		OP020444_ITS	Soil and water	Iraq	GenBank
*Lecanicillium aphanocladii* IFM 64743		LC553279_ITS LC553289_SSU LC553284_LSU LC553294_Tef1α	Tengu‐no‐Mugimeshi soil	Japan	^68^
*Lecanicillium aphanocladii* fki‐9593			Tengu‐no‐Mugimeshi soil	Japan	^69^
*Lecanicillium aphanocladii* IHEM:26758		OW985864_ITS	Environment (rubber carpet)	Belgium	GenBank
*Lecanicillium aphanocladii* 170915GAR11K1		MW826143_ITS	Environment (air)	Spain	^70^
*Lecanicillium aphanocladii* voucher CML 2970		KR261451_ITS	Environment (air)	Brazil	^71^
*Lecanicillium aphanocladii* sample759		LC745692_ITS	Environment (house dust)	Japan	GenBank
*Lecanicillium aphanocladii* KBP F‐23		PQ606631_ITS	Environment (indoor air)	Russia	GenBank
*Lecanicillium aphanocladii* IHEM:03485		OW983161_ITS	Environment (indoor air)	Belgium	GenBank
*Lecanicillium aphanocladii* QH69		JF911775_ITS	Environment (lake water)	China	GenBank
*Lecanicillium aphanocladii* GS73		MN511328_ITS	Environment (sawdust)	South Korea	GenBank
*Lecanicillium aphanocladii* BN35		MH484024_ITS	Envrionment (air)	China	GenBank
*Lecanicillium aphanocladii* Z06		KT189155_ITS	Envrionment (crude oil and wax)	China	GenBank
*Lecanicillium aphanocladii* NNIBRFG2172		OK090967_ITS OK090991_LSU OK235317_Tef1α	Envrionment (freshwater foam)	South Korea	^72^
*Lecanicillium aphanocladii* IHEM:28037		OU989389_ITS	Bat fur (*Plecotus auritus*)	Belgium	GenBank
*Lecanicillium aphanocladii* AUMC11913			Contaminated potato dextrose agar (PDA) plate	Egypt	^73^
*Lecanicillium aphanocladii* wb293	*Aphanocladium aranearum*	AF455489_ITS	Human patients	Austria	^74^
*Lecanicillium aphanocladii* wb578	*Aphanocladium aranearum*	AF455405_ITS	Human patients	Austria	^74^
*Lecanicillium aphanocladii* Tr247		PP564874_ITS PP729101_Tef1α	Human patients	Turkey	Genbank
*Lecanicillium aphanocladii* D05		MH346472_ITS	Human patients	Brazil	^75^
*Lecanicillium aphanocladii* IHEM:19176		OW987919_ITS	Human patients	Belgium	GenBank
*Lecanicillium aphanocladii* CBS 165.45	*Aphanocladium aranerum Aphanocladium album*			The Netherlands	^22^
*Lecanicillium aphanocladii* CBS 458.82	*Aphanocladium album*		Not reported	South Africa	^22^
*Lecanicillium aphanocladii* ARSEF 6433	*Aphanocladium album*		Not reported		^35^, ^36^, ^37^
*Lecanicillium aphanocladii* SJL10.5–3		PP467451	Not reported	China	GenBank
*Lecanicillium aphanocladii* HBU‐2019‐225		ON077432_ITS	Not reported	China	GenBank
*Lecanicillium aphanocladii* MB#144303	*Aphanocladium aranearum*		Not reported		^73^
*Lecanicillium aphanocladii* MB#308804	*Aphanocladium aranearum*		Not reported		^73^
*Lecanicillium aphanocladii* N14			Not reported		GenBank
*Lecanicillium aphanocladii* BEOFB4710m		OL336789_ITS	Not reported	Serbia	GenBank

The formerly named *Aphanocladium album* MX95 is a known parasite of phytopathogenic fungi and has been extensively studied as a possible agent for plant protection (patent MI2006A000503). Nevertheless, the species identity of MX95 is not clearly defined. As such, we conducted a sequence comparison for some key genes to determine the species identity of MX95. In 2023, a complete genome sequence of MX95 was published in GenBank (Accession GCA_029218145.1; University of Milan, Italy). Similarly, in the same year a genome assembly for *L. aphanocladii* strain MBC 099 was also published by USDA‐ARS‐NCAUR (GenBank Accession GCA_030411675.1). MX95 was initially isolated from eggplant in Italy and seems to have been reported first by Ciccarese *et al*.,^77^ whereas *L. aphanocladii* MBC 099 was isolated from an aphid in France. To compare these two fungal strains we extracted equivalent sequences from the available genomes of fungal strains of MX95 and *L. aphanocladii* MBC 099. The hypothetical non‐ribosomal peptide synthetase (NRPS) gene (GenBank Accession KAJ6780765) and the internal transcribed spacer (ITS), small‐subunit rRNA (SSU) and large‐subunit rRNA (LSU) sequences were the primary focus of analysis. The similarity of the NRPS gene sequence within MX95 and *L. aphanocladii* MBC 099 was compared. The equivalent ITS, SSU and LSU sequences from these two strains were also compared with other strains of *Aphanocladium album* and *L. aphanocladii* selected from GenBank.

Phylogenetic analysis of ITS sequences (Fig. 1) showed that MX95 was in the same clade as *L. aphanocladii* CBS101286 (GenBank Accession AJ292430) but was in a different clade to the *Aphanocladium album* strain CBS 401.70 (GenBank Accession AJ292401). There were similarities among sequences of MX95 and *L. aphanocladii* in the SSU (Fig. 2(A)) and LSU (Fig. 2(B)) genes. The SSU sequence of strain MX95 showed higher similarity with known *L. aphanocladii* strains, such as CBS797.84 and MBC 009 with GenBank Accessions KM283763 and GCA_030411675.1, respectively, but was dissimilar to *Aphanocladium album* CBS401.70 with GenBank Accession AF339568 (Fig. 2(A)). For the LSU region comparisons, strain MX95 showed 100% similarity with sequences of *L. aphanocladii* strains MBC 099 (GenBank Accession GCA_030411675.1), CBS797.84 (GenBank Accession KM283787), IFM64743 (GenBank Accession LC553284) and Bx‐Ljb (GenBank Accession OR206378), among others. However, it was distinct from *Aphanocladium album* strain CBS411.34 with GenBank Accession MH867096 at the C‐terminal region (Fig. 2(B)). Most interestingly, the hypothetical NRPS protein in MX95 (KAJ6780765), which codes 6958 amino acids (20 877 bp nucleotides), showed > 98% similarity to the NRPS protein of *L. aphanocladii* MBC 099 (contig JAPERO010000154 of genome GCA_030411675), for both amino acid and nucleotide sequences.

This molecular evidence suggests that MX95 is likely to be a strain of *L. aphanocladii*. Therefore, the current species designation of MX95 as *Aphanocladium album* is probably incorrect. The strain MX95 has been studied extensively in previous research which suggests that *L. aphanocladii* strains are likely to be effective against several plant diseases and plant parasitic nematodes (see more discussions later).

## 4 GEO‐DISTRIBUTION AND HOST DIVERSITY OF *L. APHANOCLADII*

*Lecanicillium aphanocladii* is a cosmopolitan fungus and is widely distributed across Asia, Europe, Africa and the Americas (Table 1). This fungus has been reported to be vulnerable to heat, with conidia failing to germinate at 45 °C,^35^ and sensitive to ultraviolet (UV) light as it exhibits lower tolerance to UV‐B radiation compared to other entomopathogenic fungi such as *Metarhizium anisopliae*.^35^, ^37^, ^78^, ^79^

The wide host range of *L. aphanocladii* includes insects in the orders Hemiptera, Lepidoptera and Coleoptera. *Lecanicillium aphanocladii* has also been reported in association with mosquitoes (Culicidae),^80^ greenhouse whitefly *Trialeurodes vaporariorum*,^81^ silkworm *Bombyx mori*,^82^ lace bug *Leptopharsa heveae*,^36^ aphids *Rhopalosiphum* spp.,^28^ cyst nematodes *Globodera* spp.,^43^ spider *Stenoterommata platensis*,^38^ cockchafer beetle *Melolontha melolontha*,^42^ tobacco moth *Ephestia elutella*,^62^ flour moth *Ephestia kuehniella*,^29^ meadow spittlebug *Philaenus spumarius*,^22^ western flower thrips *Frankliniella occidentalis*,^83^ pine bark beetle *Pseudips mexicanus*,^40^ horse‐chestnut leaf miner *Cameraria ohridella* and lime leaf miner *Phyllonorycter* (*Lithocolletis*) *issikii*,^26^, ^27^ and pine sawyer beetle *Monochamus alternatus*.^39^ This demonstrates that *L. aphanocladii* infects a broad range of insect hosts.

About 36% of all the reported strains of *L. aphanocladii* in Table 1 were isolated from plants. These host plants of *L. aphanocladii* range from agricultural to forest species. Several *L. aphanocladii* isolates from plant hosts were reported as endophytes that caused no visible disease, for example, *Hemidesmus indicus*,^52^ *Huperzia serrata*,^49^ *Ageratina adenophora* (NCBI submission), *Magnolia denudata* cv. Hailuo,^53^ *Bupleurum chinense*,^55^ *Picea mariana* ^54^ and *Zea mays* ^56^ (Table 1). Research on endophyte–insect–plant interactions is critical to advance our knowledge of entomopathogenic fungi as a sustainable pest management approach.^84^ Further exploration and characterisation of a wider range of endophytic *L. aphanocladii*, particularly those with potent entomopathogenic properties against important agricultural pests and diseases, are required in future studies.

There are several reports that *L. aphanocladii* has been isolated from different species of mushrooms (Table 1), causing diseases such as cobweb and spotting diseases in *Agaricus bisporus* in Europe,^30^ as well as recently reported cobweb disease in *Schizophyllum commune*,^58^ and rot diseases in *Sparassis latifolia*,^60^ *Tremella fuciformis*,^59^ and *Morchella sextelata* ^85^ in China. *Lecanicillium aphanocladii* is also a fungicolous species that associates with *Agaricus bitorquis*, *Podosphaera* (*Erysiphe*) *fuliginea*, *Podosphaera pannosa*, *Uromyces savulescui*,^31^ *Puccinia* spp,^86^, ^87^ and *Altenaria tenius* (PDD 42012, NZFungi database, Landcare Research, New Zealand).

*Lecanicillium aphanocladii* has also been isolated from soil samples (11%; Table 1), environmental materials (12%) and other types of specimens (18%), including unknown sources. Notably, a few strains have been isolated from human nasal mucus,^74^ a dermatophyte infection,^75^ and bat fur (GenBank Accession OU989389). While fungi, in general, can be opportunistic pathogens in severely immunocompromised humans, due to its specific entomopathogenic characteristics *L. aphanocladii* is considered to have low or negligible infection risk to mammals and humans. Related species such as *L. lecanii* are considered safe to humans and non‐target organisms, and have a favourable environmental risk profile.^88^

## 5 BIOPESTICIDE ACTIVITY OF *L. APHANOCLADII*

Several *L. aphanocladii* strains have been reported to be active against a range of insect pest species, including aphids that cause damage in many agricultural crops.^89^, ^90^ For instance, the corn leaf aphid (*Rhopalosiphum maidis*) is a common pest of maize and other related crops globally.^91^ Different species of entomopathogenic fungi from the phyla Ascomycota and Entomophthoromycota are associated with aphids, but only a few of these are specific for aphids.^28^ *Lecanicillium aphanocladii* is one of the most important hypocrealean pathogens of aphids. In a glasshouse trial studying barley yellow dwarf virus on cereal crops in Iran, Zare and Mohammad^28^ observed that aphids of *Rhopalosiphum maidis*, *Rhopalosiphum padi*, *Schizaphis graminum* and *Sitobion avenae* were heavily colonised by *L. aphanocladii*, leading to mortality in 7–10 days. *Lecanicillium aphanocladii*‐Hu 1 has also been patented as a biocontrol agent against green peach aphid *Myzus persicae* (Sulzer) (Patent CN119320701A^32^) in China. The high pathogenicity of *L. aphanocladii* to aphids makes it a potential biological control agent to prevent transmission and dispersal of plant‐pathogenic viruses.^28^ Studies on other species of *Lecanicillium*, such as *L. attenuatum*, *L. lecanii*, and *L. longisporum*, have also demonstrated high efficacy against different aphid species.^6^, ^90^

Furthermore, the leaf miner moths *Cameraria ohridella* and *Phyllonorycter* (*Lithocolletis*) *issikii* are pests of horse chestnut (*Aesculus hippocastanum*) and small‐leaved linden (*Tilia cordata*) in Lithuania. *Lecanicillium aphanocladii* was reported to be the most common species recovered from larval cadavers of the leaf miners, with infection frequency ranging from 30% to 42% for *Cameraria ohridella* and *Phyllonorycter issikii*, respectively.^26^, ^27^ The *L. aphanocladii* isolates showed high pathogenicity in a comparative laboratory bioassay by Nedveckytė *et al*.^26^ Indeed, *L. aphanocladii* was the most virulent fungal species against *Cameraria ohridella*, causing 77% mortality in eggs, which was 1.8‐, 2.0‐, and 2.8‐fold higher than *Beauveria bassiana*, *Isaria fumosorosea* or *L. psalliotae*, respectively. Nedveckytė *et al*.^26^ reported that *L. aphanocladii* was associated with horse chestnut foliage and showed that it was common on leaf surfaces with powdery mildew disease (*Erysiphe flexuosa*).

*Lecanicillium aphanocladii* strain F28’ was found in association with insect pests of olive trees (*Olea europaea*),^29^ isolated from olive fly (*Bactrocera oleae*) as well as from olive psyllid (*Euphyllura olivine*) cadavers collected from Tunisian olive groves. Strain F28’ was found to be highly pathogenic to *Ephestia kuehniella* (Mediterranean flour moth) fourth‐instar larvae resulting in 93% mortality. In addition, *L. aphanocladii* F28’ displayed no phytopathogenic activity against excised olive shoots at 28 days post‐inoculation.^29^

Bodino *et al*.^92^ isolated *L. aphanocladii* and three other entomopathogenic species, *Beauveria bassiana*, *Conidiobolus coronatus* and *Fusarium equiseti* from dead spittlebug (*Philaenus spumarius*), the key vector of *Xylella fastidiosa* a plant pathogen transmitted by *Philaenus spumarius* in Europe.^93^, ^94^ Subsequent studies showed that a blastospore formulation of *L. aphanocladii* was lethal to spittlebug nymphs (90% mortality) and also reduced adult emergence with an efficacy that was similar to a commercial insecticide based on *Beauveria bassiana*, Naturalis®.^92^ Zhou *et al*.^62^ applied conidial suspensions of *L. aphanocladii* GzuiFr‐lun1505 isolated from forest rhizosphere soil in China. Conidia were lethal to third‐instar larvae of the tobacco moth *Ephestia elutella* with recorded 78% mortality, which was similar to the mortality caused by *Isaria cateinannulata* (79%) and *Beauveria bassiana* (65%). Rangel and Correia^36^ compared the virulence of *L. aphanocladii* (ARSEF 6433) with *L*. (*Verticillium*) *lecani* (ARSEF 6430, 6431 and 6432) on a rubber tree pest, lace bug (*Leptopharsa heveae*) third‐ and fifth‐instar nymphs and adults by applying conidial suspensions, at concentrations of 2.4 × 10^5^–2.4 × 10^7^ conidia per mL. At the highest concentrations *L. aphanocladii* ARSEF 6433, *L. lecani* ARSEF 6430 and 6432 showed median lethal time (LT50) values of 2.6, 2.6 and 3.2 days, respectively. Although *L. aphanocladii* shows good pathogenicity against a range of pests its bioactivity is sometimes lower than some other *Lecanicillium* species. For example, mortality to Western flower thrips (*Frankliniella occidentalis*), a pest of numerous crops, was lower than species of *L. attenuatum*, *Lecanicillium cauligalbarum* and *Lecanicillium araneogenum*,^83^ indicating that the pathogenicity of *L. aphanocladii* differs depending on the target pest.

There are some more recently identified hosts of *L. aphanocladii* such as *Pseudips mexicanus* (tree bark beetle),^40^ *Melolontha melolontha* larvae (cockchafer beetle)^42^ and *Monochamus alternatus* (pine sawyer beetle),^39^ which need to be further studied to understand the impact of *L. aphanocladii* on coleopteran pests.

## 6 FUNGICIDAL AND NEMATICIDAL ACTIVITY OF *L. APHANOCLADII*

Generally, the bioactivity of biological control agents comes in different forms where they act as antagonists against a variety of organisms, including nematodes, fungi and bacteria. *Lecanicillium aphanocladii* is known as a fungicolous species attacking *Podosphaera fuliginea and Podosphaera pannosa*,^28^ *Puccinia* spp.,^95^ and *Limonium* rust.^96^ Earlier studies on *L. aphanocladii* strains against those pathogenic fungi have been reviewed.^28^, ^31^ The use of *L. aphanocladii* as a biocontrol agent against phytopathogenic fungi and phytoparasitic nematodes has been applied practically,^97^, ^98^ however most studies have focused on strain MX95 (under the name *Aphanocladium album*) (as discussed later). Studies on other *L. aphanocladii* strains that have activity against phytopathogens are needed in the future, particularly the strains that show pesticidal activity. There are only two reports of *L. aphanocladii* being antagonistic against bacteria.^44^, ^68^ Higo *et al*.^68^ reported that *L. aphanocladii* (FKI‐9593 = IFM 64743) had weak antibacterial activity against *Proteus vulgaris* NBRC 3167 and Zhou *et al*.^44^ showed that the ethyl acetate extract from *L. aphanocladii* AF15 was active against Gram‐positive pathogenic bacteria, including methicillin‐resistant *Staphylococcus aureus*, *Staphylococcus epidermidis*, vancomycin‐resistant *Enterococcus faecalis* and *Streptococcus agalactiae*. In addition, it has been reported that *Bacillus subtilis* A9 from morel mushroom (*Morchella esculenta*)^57^ and *Pantoea* spp. from maize kernels^56^ have antagonistic effects on *L. aphanocladii*.

The *L. aphanocladii* MX95 was able to inhibit the growth and sporulation of several foliar pathogens including powdery mildew and rust and some soil‐borne plant pathogens, such as nematodes.^33^, ^87^, ^98^, ^99^, ^100^ MX95 was also a potential biological limiter of the Esca and root rot fungi in grapevine.^98^ An *in vitro* anti‐fungal test showed that MX95 effectively prevented mycelia and conidia spread from colonising pathogenic *Fomitiporia mediterranea*, *Phaeoacremonium minimum* and *Phaeomoniella chlamydospora* on detached grapevine canes. MX95 also controlled postharvest rot diseases of grape preharvest and postharvest,^101^, ^102^ and demonstrated some suppression against *Golovinomyces lycopersici*, the causal agent of powdery mildew in tomato and squash, and *Podosphaera fusca* causing powdery mildew in cucumber.^77^, ^103^ In tomato, MX95 was reported to limit corky root disease caused by *Pseudopyrenochaeta lycopersici*.^100^, ^104^ Others applied MX95 to soil by sub‐surface irrigation to reduce the severity of corky root in tomato, whereas applications to eggplants reduced wilting and vascular discoloration caused by *Verticillium dahlia*.^105^ When MX95 was applied to melon plots, fusarium wilt was significantly reduced, compared to plots treated with the chemical fumigant Dazomet.^106^ A recent study showed that in laboratory dual culture tests, MX95 completely suppressed growth of the pathogenic fungi *Phytophthora nicotianae* and *Sclerotinia minor*, and reduced the mycelial growth of *Fusarium solani* and *Sclerotinia sclerotiorum* by 40% and 20%, respectively.^33^ Research has also shown that the application of MX95 significantly increased rhizosphere microbial populations in tomato plants.^97^, ^99^

The potential nematocidal activity of MX95 against the root‐knot nematode *Meloidogyne* spp. in infected plants was investigated.^97^, ^99^, ^101^, ^107^ The MX95 treatments, applied by sub‐surface irrigation were simultaneously effective against the soil borne pathogen *Pseudopyrenochaeta lycopersici* and the root‐knot nematode *Meloidogyne incognita*.^107^ MX95 decreased root gall formation by *Meloidogyne javanica* in infected tomato plants.^99^ The MX95 treatment effects on the rhizosphere microbiome were also analysed by a metabarcoding procedure, which revealed that MX95 significantly decreased root gall severity index and soil nematode population of *Meloidogyne javanica*.^97^ This research suggested that MX95 has strong potential as a bionematicide, although no significant effects on *Meloidogyne javanica* were reported for other *L. aphanocladii* strains.^108^

## 7 BIO‐INSECTICIDAL AND BIO‐FUNGICIDAL METABOLITES OF *L. APHANOCLADII*

The efficacy of *L. aphanocladii* MX95 against phytopathogens and nematodes may be linked to the production of hydrolytic enzymes such as proteases, glucanases, and several chitinases involved in the cell wall degradation of fungal phytopathogens.^97^ Secretion of chitinase A by MX95 was identified as an extracellular, chitin‐inducible enzyme that is essential for its fungicidal activity.^97^ The *in silico* functional annotation of the genome of MX95 allowed the identification of 46 genes encoding chitinolytic enzymes of the GH18 (26 genes), GH20 (eight genes), GH75 (eight genes), and GH3 (four genes) families.^97^ These enzymes have either endochitinase or exochitinase activity and play a diversity of roles including interactions with other fungi, the degradation of exogenous chitin for nutrient acquisition, cell wall remodelling, and hyphal growth.^97^, ^109^ Chitinolytic enzymes are responsible for the total or partial disruption of the cell walls of many phytopathogenic fungi and parasites^97^, ^110^ and are also necessary for hyphal penetration of the cuticle of insects, nematodes or spiders.^17^, ^111^, ^112^ Other enzyme complexes, such as proteases and β‐glucanases, have similar roles.^17^, ^113^, ^114^, ^115^

The production of secondary metabolites by entomopathogenic fungi is critical for pathogenicity and disease development in host insects. For instance, bassianolides from *Beauveria bassiana* and destruxins from *Metarhizium* spp. contribute to the biocontrol of pests and pathogenic fungi.^116^, ^117^, ^118^, ^119^, ^120^, ^121^, ^122^, ^123^, ^124^, ^125^ However, the roles of secondary metabolites as virulence factors expressed by *L. aphanocladii* against insect pests are poorly understood. Destruxins are among the most exhaustively researched secondary metabolites of entomopathogenic fungi and have been shown to have both insecticidal and phytotoxic activities.^3^, ^120^, ^121^, ^122^, ^123^ Most destruxins and their analogues have been isolated from cultures of *Metarhizium* spp. but are less documented in other fungi,^124^ although some derivatives of destruxins have been extracted from *Beauveria felina*, *Alternaria brassicae* and *Nigrosabulum globosum*.^3^, ^120^, ^125^ The presence of destruxin‐like compounds has also been identified from *L. longisporum*, in which these metabolites were present at significantly higher concentration in stirred aerated cultures compared with static cultures.^124^

*Lecanicillium aphanocladii* has been shown to contain a hypothetical NRPS protein (GenBank Accession KAJ6780765 in strain MX95), which has sequence similarities with destruxin synthetases from various other fungi, particularly *Metarhizium* species (Table 2), suggesting that *L. aphanocladii* might have the potential to synthesise destruxin‐like compounds. Repeated extraction and analysis of destruxins from *L. longisporum* indicated that the production of destruxins was more variable compared to the production by *Metarhizium* spp., indicating that additional factors probably influence destruxin synthesis.^124^

**Table 2:** Results of BLASTp search for hypothetical protein of Accession KAJ6780765 (*Lecanicillium aphanocladii* strain MX95) in National Centre for Biotechnology Information (NCBI) GenBank

Description	Scientific name	Maximum score	Total score	Query cover	*E* Value^†^	Per Ident^‡^	Acc Len^§^	Accession
Destruxin synthetase	*Pyrenophora teres* f. *teres*	5447	23 267	100%	<1e‐100	42.25	9097	CAE7020799.1
Destruxin synthetase	*Pyrenophora teres* f. *teres*	4497	15 594	99%	<1e‐100	41.82	5906	CAE7021457.1
Destruxins‐like synthetase	*Tolypocladium ophioglossoides*	3518	12 112	100%	<1e‐100	37.87	8012	KND86507.1
Destruxins synthetase	*Metarhizium anisopliae*	3457	11 836	100%	<1e‐100	37.49	7844	KAK8912138.1
Destruxin synthetase	*Metarhizium robertsii*	3451	12 692	100%	<1e‐100	37.43	7913	XP_007826232.2
Destruxin synthetase	*Metarhizium hybridum*	3448	12 640	100%	<1e‐100	37.44	7912	KID59658.1
Destruxin synthetase	*Metarhizium anisopliae*	3447	12 613	100%	<1e‐100	37.44	7894	KFG77713.1
Destruxin synthetase	*Metarhizium brunneum*	3443	12 641	100%	<1e‐100	37.39	7888	XP_065987973.1
Destruxin synthetase	*Metarhizium brunneum*	3442	12 639	100%	<1e‐100	37.39	7912	KID61226.1
Destruxins synthetase	*Beauveria felina*	3440	14 675	100%	<1e‐100	37.19	8002	QTW21125.1
Destruxin synthetase	*Metarhizium guizhouense*	3436	12 683	100%	<1e‐100	37.29	7910	KID82708.1
Destruxin synthetase	*Metarhizium brunneum*	3414	12 579	100%	<1e‐100	37.18	7887	KAK9446167.1
Destruxin synthetase	*Metarhizium anisopliae*	3413	12 545	100%	<1e‐100	37.17	7907	KAF5121803.1
Destruxin synthetase	*Cladobotryum mycophilum*	3359	13 243	100%	<1e‐100	41.03	7989	KAK5991451.1
Destruxin synthetase	*Metarhizium majus*	3251	12 272	100%	<1e‐100	39.99	7904	KID94891.1

^†^ The *E* values calculated by BLASTp fall below the display threshold. We use ‘<1e‐100’ to replace value of ‘0’ that was generated by BLASTp.

^‡^ Percentage identity.

^§^ Accession length.

Other pigment‐based compounds such as oosporein, orevactaene and dihydrotrichodimerol have been identified in extracts of *L. aphanocladii* (CML2970).^71^ The polyketide metabolite oosporein was also identified from *L. aphanocladii* strain FKI‐9593 (=IFM 64743).^69^ These results suggest that certain strains of *L. aphanocladii* might serve as novel sources of pigments that could have industrial and biological applications. Oosporein has antifungal activities,^71^, ^126^ including against *Phytophthora infestans*,^127^ *Rhizoctonia solani*, *Botrytis cinerea* and *Pythium ultimum*, and is reported to inhibit the proliferation of tumour cell lines.^128^ Dihydrotrichodimerol reduces feeding in the aphid *Schizaphis graminum*, one of the most important pests of cereal crops, suggesting possible applications in the control of this pest.^129^ In addition, water soluble secondary metabolites synthesised by *L. aphanocladii* (CML2970) have been reported to be effective in inhibiting snake venom‐induced proteolysis and phospholipase A_2_ activity, which may be of use in the development of pharmaceuticals of medical interest.^64^ Several structurally characterised secondary metabolites from *Lecanicillium* spp. have been reported including indolosesquiterpenoids,^130^ phenopicolinic acid analogues,^131^ tetracyclic diterpenoid,^132^ pregnanes,^133^ cyclic lipodepsipeptides,^134^ and lecanicillolide^49^, ^135^ along with several related compounds, spiciferone,^136^ dihydrospiciferone^137^ and butoxyl‐spiciferin.^138^ These compounds are primarily of interest in biomedical research and drug discovery, especially for their potential anti‐cancer, anti‐inflammatory, and antimicrobial activities. However, these metabolites have not been reported in *L. aphanocladii*.

## 8 SUMMARY

Entomopathogenic fungi hold immense potential as biological control agents against insect pests of important agricultural and horticultural crops. The unexplored diversity of entomopathogenic fungi underscores the need for continued bioprospecting and evaluation of novel strains. This review indicates that several *L. aphanocladii* strains have great potential to be developed as multipurpose biocontrol agents that are active against several insect pests, plant diseases and plant parasitic nematodes. Developing commercial biocontrol agents based on *L. aphanocladii* would provide eco‐friendly products that could safeguard agricultural production amidst regulatory pressures to reduce synthetic pesticide use. However, some considerations for future studies remain.

The virulence of *L. aphanocladii* against insect pests has largely been characterised under laboratory conditions with limited field trials. This significantly restricts the broader application of this entomopathogen in pest control. Further studies on fungal application and field efficacy in the natural environment are required. Under field conditions, fungal biopesticides are usually applied using an inundative approach, which often involves exposure of the fungal conidia to unfavourable humidity, temperature and solar radiation conditions. These abiotic factors reduce the persistence and efficacy of fungal products. Formulation technologies and the application of *L. aphanocladii* need to be considered which maximise biocontrol efficacy by increasing the field persistence, improve shelf life, facilitate product application and increase the virulence of these pathogens.

For *L. aphanocladii*, some difficulties may occur during mass conidia production *in vitro* and when considering the stability of propagules for storage and formulation. Mass production methods and advances in the formulation of microbial insecticides have been reviewed previously.^139^, ^140^ Large scale applications of blastospores in aqueous suspensions have been used for *Beauveria brongniartii* against European cockchafer, *Melolontha melolontha*.^141^ Successful inoculation of fungal blastospores on plant seeds to produce mycelium and aerial conidia against insect pests has also been reported in other entomopathogenic fungi, such as *Beauveria brongniartii* on barley seeds.^141^ These techniques may be applicable to *L. aphanocladii*. Development of fungal consortia using combined *L. aphanocladii* strains against various insect pests and phytopathogens may favour the creation of products with a wider spectrum of action than individual strains. Continued exploration and characterisation of a wider range of endophytic *L. aphanocladii*, particularly those with potent entomopathogenic properties, is another important objective worth pursuing in future studies. In addition to insect pest control, the ability of endophytic *L. aphanocladii* to colonise plants will add a new dimension to its utilisation as a biological agent.

Genetic manipulation of biocontrol fungal agents is technically feasible and could provide useful strategies to either increase fungal virulence or enhance resistance to different stress factors. Increases in the pathogenicity of some entomopathogenic fungi has been achieved through genetic interventions involving the incorporation of genes encoding neurotoxic peptides, proteases, antimicrobial peptides, chitinases for cuticle degradation, and peptides that influence insect physiology.^142^ Nevertheless, widespread adoption is currently limited by strict and comprehensive regulatory frameworks, which are driven by safety concerns, potential ecological risks, and public perception issues.

The secondary metabolites from entomopathogenic fungi represent an additional aspect of their biocontrol potential and pest control efficacy. However, the roles of secondary metabolites produced by *L. aphanocladii* are poorly understood. A deeper understanding of the range of compounds produced and their biosynthetic pathways are likely to provide insights into their interactions with insects, plant pathogens and crop plants. In summary, evidence suggests that developing cost‐effective fit‐for‐purpose biological control agents from *L. aphanocladii* could lead to effective replacements for current synthetic chemical control options with numerous potential benefits for modern agricultural production.

## AUTHOR CONTRIBUTIONS

Qianhe Liu: writing – original draft, writing – review and editing. Richard Johnson: writing – review and editing. Kwasi Adusei‐Fosu: writing – review and editing.

## CONFLICT OF INTEREST

The authors declare that they have no conflict of interest to disclose.

## Data Availability

Data sharing is not applicable to this article as no new data were created in this study.
